# Multi-distance frequency-domain optical measurements of coherent cerebral hemodynamics

**DOI:** 10.3390/photonics6030083

**Published:** 2019-07-26

**Authors:** Giles Blaney, Angelo Sassaroli, Thao Pham, Nishanth Krishnamurthy, Sergio Fantini

**Affiliations:** Tufts University, Department of Biomedical Engineering

**Keywords:** near-infrared spectroscopy, diffuse optical imaging, frequency-domain, brain, cerebral blood flow, cerebral blood volume, coherent hemodynamics, phasor

## Abstract

We report non-invasive, bilateral optical measurements on the forehead of five healthy human subjects, of 0.1 Hz oscillatory hemodynamics elicited either by cyclic inflation of pneumatic thigh cuffs, or by paced breathing. Optical intensity and the phase of photon-density waves were collected with frequency-domain near-infrared spectroscopy at seven source-detector distances (11-40 mm). Coherent hemodynamic oscillations are represented by phasors of oxyhemoglobin (**O**) and deoxyhemoglobin (**D**) concentrations, and by the vector **D**/**O** that represents the amplitude ratio and phase difference of **D** and **O**. We found that, on average, the amplitude ratio (|**D**/**O**|) and the phase difference (∠(**D**/**O**)) obtained with single-distance intensity at 11-40 mm increase from 0.1 and −330°, to 0.2 and −200°, respectively. Single-distance phase and the intensity slope featured a weaker dependence on source-detector separation, and yielded |**D**/**O**| and ∠(**D**/**O**) values of about 0.5 and −200°, respectively, at distances greater than 20 mm. The key findings are: (1) single-distance phase and intensity slope are sensitive to deeper tissue compared to single-distance intensity; (2) deeper tissue hemodynamic oscillations, which more closely represent the brain, feature **D** and **O** phasors that are consistent with a greater relative flow-to-volume contributions in brain tissue compared to extracerebral, superficial tissue.

## 1. Introduction

Non-invasive optical measurements of the brain aim to extract functional and physiological information from data collected using light sources and optical detectors placed on the scalp [1]. Two active areas of research that spurred commercial and clinical translation include functional brain imaging [2,3] and cerebral oximetry [4,5]. One common problem in non-invasive optical measurements of the brain is the confounding contribution from superficial, extracerebral tissue (scalp, skull, dura mater; subdural, arachnoid, and subarachnoid spaces) through which all detected photons must propagate. A number of studies have investigated methods to correct for extracerebral tissue contributions [6–9]. Alternatively, the differential approach of slopes, i.e. gradients of optical signals versus source-detector separation, was found to be weakly sensitive to a uniform superficial layer that is a few millimeters thick [10]. It is also relevant to observe that quantities measured in Frequency-Domain (FD) and Time-Domain (TD) spectroscopy, namely the phase of photon-density waves and late-time-gate photons, carry a stronger sensitivity to deep tissue than Continuous-Wave (CW) measurements [11,12]. Furthermore, measurements performed at larger source-detector separations generally sense broader and deeper tissue volumes.

The vast majority of non-invasive optical measurements of the human brain relies on hemodynamic, metabolic, and/or oxygenation changes. An important class of such temporal changes are hemodynamic oscillations that may occur spontaneously [13–18] or that may be elicited in a number of different ways [19–21]. Diffuse optical measurements with Near-InfraRed Spectroscopy (NIRS) result in dynamic sensing of the tissue concentrations of both oxyhemoglobin ([HbO_2_], or *O*) and deoxyhemoglobin ([Hb], or *D*), thus providing a detailed characterization of hemodynamic oscillations. For example, the relative phase of cerebral *O* and *D* oscillations has been reported to correlate with brain development in infants [18], sleep stage in healthy adults [16], or with the presence of obstructive carotid disease [19]. Recently, we have introduced a mathematical framework for a quantitative characterization of cerebral hemodynamic oscillations measured with NIRS, referred to as Coherent Hemodynamics Spectroscopy (CHS) [22,23].

In this work, we report FD NIRS measurements on the forehead of healthy human subjects, in which we induced hemodynamic oscillations at a frequency of 0.1 Hz either by a cyclic inflation-deflation of pneumatic thigh cuffs, or by paced breathing. These are typical protocols for CHS [22,23], which aims to investigate the dynamic cerebrovascular response to systemic arterial pressure changes. The optical measurements included intensity and phase at source-detector separations ranging from 11-40 mm. The goal of this study is to investigate the relationship between hemodynamics measured using different kinds of FD NIRS data (intensity and phase) at different source-detector separations (which probe different tissue depths), as well as intensity slope data collected from different sets of source-detector separations, which also probe different tissue depths while also being less sensitive to uniform changes in a superficial tissue layer. The rationale for this study is that sets of NIRS data that feature different levels of sensitivity to deeper versus superficial tissue may provide indications on cerebral versus extracerebral tissue hemodynamics, thus helping to address the issue of extracerebral tissue contamination in NIRS signals. Therefore, this study explores avenues for more specific NIRS sensitivity to brain tissue, with a specific focus on elicited cerebral hemodynamic oscillations.

## 2. Methods

### 2.1. Human Subjects, Near-InfraRed Spectroscopy (NIRS) Instrument, and Measurement Protocol

**Figure 1.**
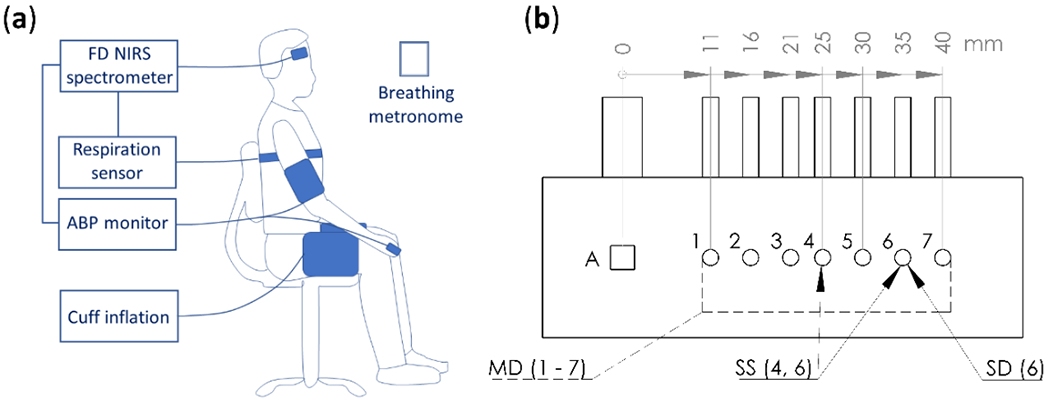
(**a**) Diagram of experimental setup showing seated subject with Frequency-Domain (FD) Near-InfraRed Spectroscopy (NIRS) probes, strain gauge respiration sensor, finger plethysmography to monitor Arterial Blood Pressure (ABP), pneumatic thigh cuff inflations inducing ABP oscillations, and a breathing metronome used in the paced-breathing portion of the protocol. (**b**) Schematic drawing of probe geometry. Detector shown as a square and labeled A, sources shown as circles and labeled 1-7 with distances ranging from 11-40 mm. Multi-Distance (MD) measurements utilized all sources to find absolute optical properties. Summary Single-Slope (SS) and Single-Distance (SD) analysis was done for all sources (paired sources spaced ~10 mm for SS, Section 3.2). Thorough analysis of SS data was done using sources 4 and 6 (25 mm and 35 mm), and thorough analysis of SD data was done using source 6 (35 mm, Section 3.3).

Data were collected on five healthy human subjects, three females and two males. The youngest subject was 25 years old and the oldest 53 years old. Experiments were repeated ten times on each subject, except subject 2 for whom measurements were repeated five times. FD NIRS data were collected at two wavelengths, 690 nm and 830 nm, and at a modulation frequency of 140.625 MHz using a commercial instrument (ISS Imagent, Champaign, IL, USA). Two optical probes were used, each featuring one detector fiber bundle and seven pairs of source optical fibers (each pair delivering light at two wavelengths), with source-detector distances ranging from 11-40 mm (Figure 1(b)). The two optical probes were placed on the right and left sides of the subject’s forehead. Arterial Blood Pressure (ABP) was measured using finger plethysmography (CNSystems CNAP Monitor 500, Graz, Austria). ABP was measured continuously on the subject’s left index or middle finger, and it represents instantaneous ABP values, which thus reflect systolic maxima and diastolic minima. We do not translate instantaneous ABP measurements into Mean Arterial Pressure (MAP), but we rather apply suitable filters to isolate frequency components of interest. Respiration was measured using a band with strain gauge placed around the subject’s chest (Ambu Respiration Belt, Ballerup, Denmark) (Figure 1(a)).

ABP oscillations were induced at a frequency of 0.1 Hz using two protocols. The first protocol involved a cyclic inflation and deflation of two pneumatic cuffs placed on each of the subject’s thighs (Hokanson CC17, Bellevue, WA, USA). The cuffs inflated to a maximum pressure of 200 mmHg regulated by an automated cuff inflation system (Hokanson E20, Bellevue, WA, USA). The pressure of the cuffs was measured using a pressure transmitter (Dwyer 626 Series, Michigan City, IN, USA) and recorded. The second protocol involved paced breathing. Subjects were visually prompted for when to inhale and exhale using a custom metronome programmed in MATrix LABoratory (Mathworks MATLAB, Natick, MA, USA) and displayed on a screen. Their respiration was monitored using the respiration belt.

**Figure 2.**
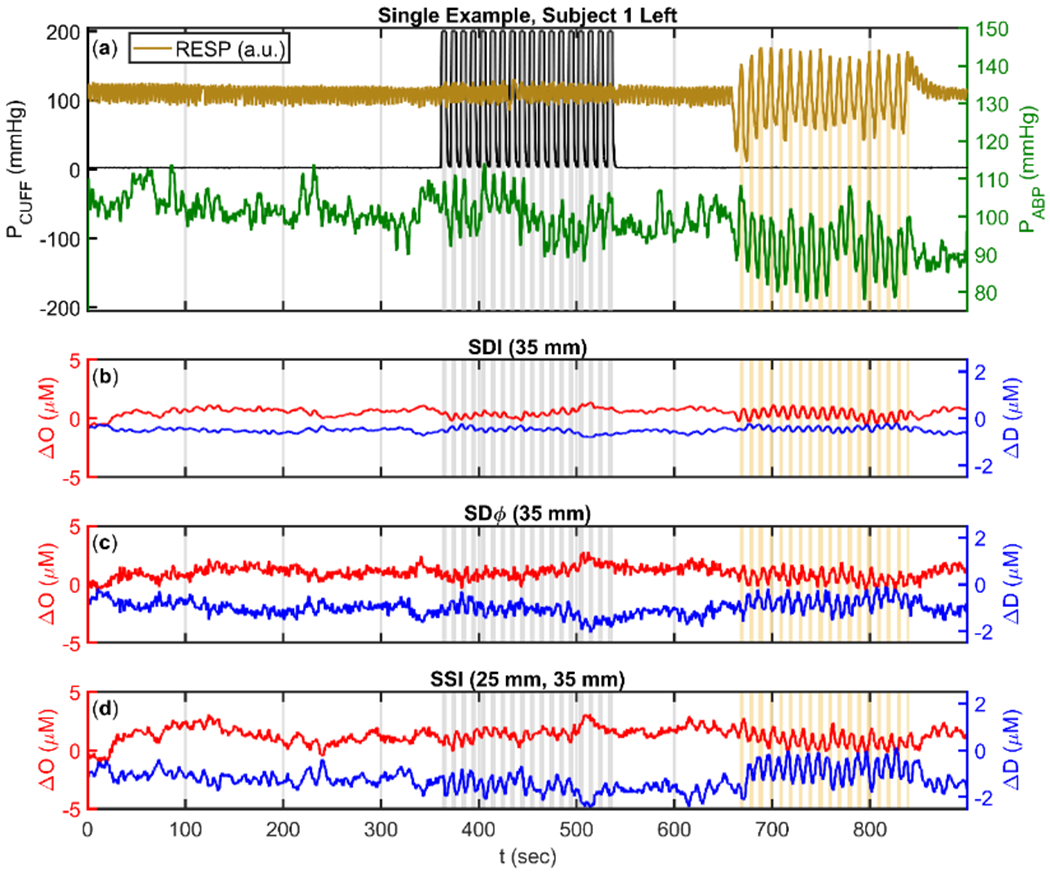
Example time (t) traces for experimental protocol. (**a**) Pneumatic CUFF pressure (P_CUFF_, Black), Arterial Blood Pressure (ABP) (P_ABP_, Green), RESPiration signal (RESP, Mustard). (**b**) Changes in Oxyhemoglobin (ΔO, Red, mean at 0.5 μM) and Deoxyhemoglobin (ΔD, Blue, mean at −0.5 μM) measured using Single-Distance Intensity (SDI) with source 6 (35 mm, Figure 1(b)). (**c**) Changes in ΔO (Red, mean at 1 μM) and ΔD (Blue, mean at −1 μM) measured using Single-Distance phase (SDφ) with source 6 (35 mm, Figure 1(b)). (**d**) Changes in ΔO (Red, mean at 1.25 μM) and ΔD (Blue, mean at −1.25 μM) measured using Single-Slope Intensity (SSI) with sources 4 and 6 (25 mm, 35 mm, Figure 1(b)). Note: All traces are lowpass filtered to 0.2 Hz, and shifted from zero baseline for visualization.

The full experimental protocol lasted 15 min (Figure 2(a)). This protocol consisted of five sections. First, a 6 min baseline period. Second, 3 min of cuff oscillations at 0.1 Hz. Third, a 2 min rest period. Forth, 3 min of paced breathing oscillations at 0.1 Hz. Finally, 1 min of rest. All data were collected at a sampling rate of approximately 9.9 Hz during the 15 min-long experiment. The protocol was approved by the Tufts University Institutional Review Board (IRB) and all subjects gave their written informed consent before participating in the study.

### 2.2. Measurement of Tissue Optical Properties and Hemoglobin Concentrations

In Section 2.2.1, we describe the FD Multi-Distance (MD) data for absolute measurement of optical properties (absorption and scattering). In Sections 2.2.2, 2.2.3, and 2.2.4, we describe three ways to extract changes in tissue absorption from FD NIRS data. These three methods are based on Single-Distance Intensity (SDI), Single-Distance phase (SDφ), and Single-Slope Intensity (SSI) data. For theory on these three methods see [24] and for a description and discussion of their practical application see [25]. For all methods, absolute or relative measurements of the absorption coefficients at two wavelengths were translated into corresponding measurements of tissue concentrations of *O* and *D* using their known extinction coefficients [26].

#### 2.2.1. Absolute Measurements with Multi-Distance (MD) Intensity (I) and Phase (φ)

Baseline absolute measurements of absorption and reduced scattering coefficient were obtained using MD FD NIRS data [27] at both wavelengths. Prior to the beginning of the experiment the FD NIRS probes were calibrated for Intensity (I) factors and phase (φ) offsets using an optical phantom of known optical properties. Average I and φ was calculated for each of the 7 source-detector distances (Figure 1(b)) over the 6 min baseline (Figure 2(a)). From these average values, the slopes of ln(*ρ*^2^I_0_) (which we denote as Single-Slopes (SS) as they are obtained using a single optical detector), SSI_0_, and the slope of φ_0_., SSφ_0_, versus *ρ* were found using least squares regression. Here, *ρ* is the source-detector distance and the 0 subscript denotes the temporal average over the baseline period. The absolute baseline absorption coefficient (*μ_ao_*) and reduced scattering coefficient (*μ_s0_^’^*) are given by [28]:

(1) $$\mu_{a0}=\frac{\omega }{2\upsilon }\left(\frac{\text{SS}\phi_{0}}{{\text{SSI}}_{0}}-\frac{{\text{SSI}}_{0}}{\text{SS}\phi_{0}}\right)$$

(2) $$\mu_{s0}^{\prime }=\frac{{\text{SSI}}_{0}^{2}-\text{SS}\phi_{0}^{2}}{3\mu_{a0}}-\mu_{a0}^{\prime }$$

where *ω* is the angular modulation frequency (*ω* = 2*πf_mod_*, where *f_mod_* is the modulation frequency), and *v* is the speed of light in the medium (*v* = *c*/*n*, where *c* is the speed of light and *n* is the index of refraction of the medium assumed to be 1.4). Equations (1) and (2) were used to find the absolute baseline absorption coefficient and reduced scattering coefficient for each subject, location, day, and wavelength using all 7 source-detector distances. Here the key assumption is that the medium is homogeneous and semi-infinite (i.e. it extends indefinitely beyond the surface over which the optical probe is placed). The baseline optical properties obtained in this way were used in the calculations described in the following Sections 2.2.2, 2.2.3, and 2.2.4.

#### 2.2.2. Relative Measurements with Single-Distance Intensity (SDI)

SDI changes where translated into changes in the absorption coefficient. The translation was based on the modified Beer-Lambert Law (mBLL) and the Differential Pathlength Factor (DPF) [28,29] in the semi-infinite medium geometry:

(3) $${\Delta \mu }_{a,\text{SDI}}\left(t\right)=\frac{\text{In}\left(\frac{{\text{SDI}}_{0}}{\text{SDI}\left(t\right)}\right)}{\rho \text{DPF}}≅-\frac{\frac{\Delta \text{SDI}\left(t\right)}{{\text{SDI}}_{0}}}{\rho \text{DPF}}$$

(4) $$\text{DPF=}\frac{\langle {\text{L}}_{\text{SDI}}\rangle }{\rho }≅\frac{\sqrt{{3\mu }_{s0}^{\prime }}}{\text{2}\sqrt{\mu_{a0}}}\frac{\rho \sqrt{{3\mu }_{a0}\mu_{s0}^{\prime }}}{\rho \sqrt{{3\mu }_{a0}\mu_{s0}^{\prime }}\text{+1}}$$

Equations (3) and (4) show the SDI absorption change calculation, where <*L*_SDI_> is the mean optical pathlength associated with SDI, at the distance *ρ*. Here the Δ prefix is used to denote changes from the baseline value (Section 2.2.1). Equation (3) is the general CW definition of DPF which is based on small intensity changes due to a small uniform absorption change, while Equation (4) is the expression of DPF in a semi-infinite, homogenous medium. Example time traces of Δ*O* and Δ*D* calculated with SDI can be seen in Figure 2(b).

#### 2.2.3. Relative Measurements with Single-Distance Phase (SDφ)

SDφ changes where translated into changes in the absorption coefficient as follows (Equations (5)):

(5) $${\Delta \mu }_{a,\text{SD}\phi }\left(t\right)=-\frac{\Delta \text{SD}\phi \left(t\right)}{\langle {\text{L}}_{\text{SD}\phi }\rangle }$$

where <*L*_SDφ_> is the mean optical pathlength associated with SDφ, which was calculated using baseline optical properties (Section 2.2.1) based on expressions of <*L*_SDφ_> reported in [24,25]. Example time traces of Δ*O* and Δ*D* calculated with SDφ can be seen in Figure 2(c).

#### 2.2.4. Relative Measurements with Single-Slope Intensity (SSI)

Intensity slopes obtained from a single detector multiple sources (as in this study), or a single source and multiple detectors are referred to as SS. Changes in the SSI were translated into changes in the absorption coefficient as follows [28]:

(6) $${\Delta \mu }_{\text{a,SSI}}\left(t\right)≅\frac{{\Delta \text{SSI}}^{2}}{{3\mu }_{s0}^{\prime }}$$

This calculation requires that the initial slope of ln(*ρ*^2^I) be calibrated to the slope corresponding to the baseline optical properties (Section 2.2.1). Equation (6) relies on the same assumptions as Equation (4). Example time traces of Δ*O* and Δ*D* calculated with SSI can be seen in Figure 2(d).

### 2.3. Coherence and Phasor Analysis

To perform CHS analysis, the Δ*O* and Δ*D* traces obtained with SDI, SDφ, and SSI were processed to yield the phase and amplitude of their oscillations during the subset of time intervals and frequency bands that featured coherent hemodynamics. Total hemoglobin concentration changes (Δ*T*) were calculated as the sum of Δ*O* and Δ*D*. Then, the wavelet coherence between Δ*O* and ABP, Δ*D* and ABP, and Δ*T* and ABP was found using a modified (to remove smoothing in scale) version of the MATLAB wcoherence function. Regions of the time-frequency space where two time-traces show coherence levels that are above a threshold value obtained with random surrogate data were considered to feature significant coherence [30]. The intersection of the time-frequency regions with significant coherence between Δ*O* and ABP and significant coherence between Δ*D* and ABP were considered for the analysis of the amplitude and phase of Δ*D* and Δ*O*. This coherence analysis is central to CHS as it identifies the tissue hemodynamics, in the time-frequency space, that are directly linked to the driving physiological process of interest (in this case, oscillations in ABP).

The Continuous Wavelet Transform (CWT) was used to find the phasor maps of Δ*O*, Δ*D*, Δ*T*, and ABP, which are indicated with bold-face phasor notation (**O**, **D**, **T**, and **ABP**). Next, the phasor ratio of **D**/**O** were calculated, and the average phasor ratio was found for the two protocols, thigh cuff and paced breathing for each subject. These averages were found by taking the average of only significantly coherent pixels in time-frequency space over the duration of the induced oscillation protocol and the frequency band centered at 0.1 Hz (the frequency of the induced oscillations) and with a bandwidth calculated using the half power bandwidth of a test sinusoidal signal. All wavelet analysis was done using the complex Morlet mother wavelet.

### 2.4. Amplitude and Phase of Oscillatory Hemodynamics that are Coherent with Arterial Blood Pressure (ABP)

The output of the described analysis was the average ratio of coherent phasor (**D**/**O**), for three data types (SDI, SDφ, and SSI), two protocols (cuff and breathing), two forehead locations (left and right), every measurement day (ten days for all subjects except subject 2, for which data were collected five days), and five subjects. The argument of the phasor ratio represents the phase difference, whereas the modulus represents the amplitude ratio of the two phasors considered. The SDI and SDφ analysis was done for 7 source-detector distances (Section 3.2) and thoroughly reported for source 6 (35 mm distance, Section 3.3), while the SSI method was done for five source-detector distances (at 10 mm increments, Section 3.2), and thoroughly reported for sources 4 and 6 (25 mm and 35 mm distance, Section 3.3).

### 2.5. Diffusion Theory Calculations

To guide the interpretation of the *in vivo* results, we performed theoretical calculations using diffusion theory to simulate optical signals resulting from illumination and optical collection from the surface of a semi-infinite medium with optical absorption of 0.1 cm^−1^ and reduced scattering coefficient of 12 cm^−1^. We considered oscillatory concentrations of *O* and *D* occurring in three layers to simulate layered hemodynamics in the scalp, skull, and brain cortex. The approach is to identify the simulated, layered hemodynamics properties that reproduce the experimental results, which can therefore be considered to be consistent with such layer-dependent hemodynamics.

## 3. Results

### 3.1. Absolute Optical Properties

Table 1 reports the baseline absolute optical properties, at the two wavelengths of 690 nm and 830 nm, averaged over all days and locations for the five subjects. From the absorption coefficients at two wavelengths, we have obtained absolute concentrations of *O*, *D*, and *T*, as well as hemoglobin Saturation (S) assuming a 70% water volume fraction [31]. The measured values of optical properties and hemoglobin parameters are consistent with previously reported values using a similar frequency-domain, multi-distance approach [31].

### 3.2. Coherence between Arterial Blood Pressure (ABP) and one of Oxyhemoglobin (ΔO), Deoxyhemoglobin (ΔD), and Total Hemoglobin (ΔT) Changes

**Figure 3.**
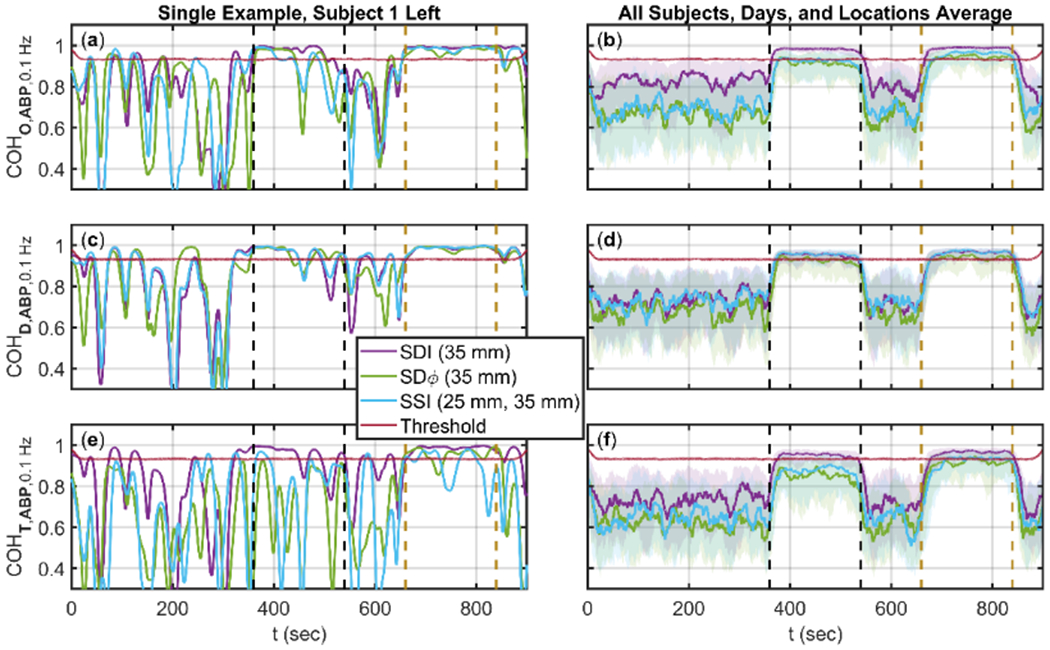
Time (t) traces of wavelet COHerence (COH) at 0.1 Hz for three phasor pairs Oxyhemoglobin (**O**) and Arterial Blood Pressure (**ABP**), Deoxyhemoglobin (**D**) and **ABP**, and Total hemoglobin (**T**) and **ABP**. Shown for three analysis methods Single-Distance Intensity (SDI, Purple), Single-Distance phase (SDφ, Green), and Single-Slope Intensity (SSI, Cyan). Threshold of significant wavelet coherence shown (Maroon). Time periods of induced cuff oscillations (Dashed Black) and induced paced breathing (Dashed Mustard) oscillations shown. (**a**)(**b**): COH between **O** and **ABP** at 0.1 Hz. (**c**)(**d**): COH between **D** and **ABP** at 0.1 Hz. (**e**),(**f**): COH between **T** and **ABP** at 0.1 Hz. (**a**)(**c**)(**e**): Example subject, day, and location. (**b**)(**d**)(**f**): Average across all subjects, days, and locations, with error regions shown as InterQuartile Range (IQR).

The temporal traces of wavelet coherence at a frequency of 0.1 Hz are reported in Figure 3 for the data pairs Δ*O* and ABP (Figure 3(a)(b)), Δ*D* and ABP (Figure 3(c)(d)), and Δ*T* and ABP (Figure 3(e)(f)), for Δ*O*, Δ*D*, and Δ*T* obtained with SDI, SSI, and SDφ data. Figure 3(a)(c)(e) are for a single measurement session on a single subject (subject 1) and single location (left), whereas Figure 3(b)(d)(f) are the average over all subjects, all days, and both locations. The time periods featuring cyclic cuff inflation and paced breathing at 0.1 Hz are indicated by black and mustard vertical dashed lines, respectively, and they feature induced hemodynamics at 0.1 Hz. During baseline, low-frequency spontaneous hemodynamic oscillations in the 0.1 Hz frequency region are present as a result of systemic physiological oscillations as well as local vasomotion [17]. Figure 3 also shows the threshold for significant coherence. Figure 3 shows that tissue hemodynamics at 0.1 Hz feature a much greater level of coherence with ABP during cyclic cuff inflation and paced breathing than during baseline. For this reason, in the following we only consider coherent hemodynamics during cyclic cuff inflation and during paced breathing. Furthermore, the coherence of SDI data is typically greater than the coherence of SSI, and SDφ data. This latter result is certainly determined, at least in part, by the greater noise of SSI, and SDφ data with respect to SDI data, but it may also result from a greater coherence between superficial hemodynamics (to which SDI data are strongly sensitive) and ABP, versus cerebral hemodynamics and ABP.

### 3.3. Deoxyhemoglobin and Oxyhemoglobin Phasor Ratio (**D**/**O**) as a Function of Source-Detector Distance for Different Measurement Methods

**Figure 4.**
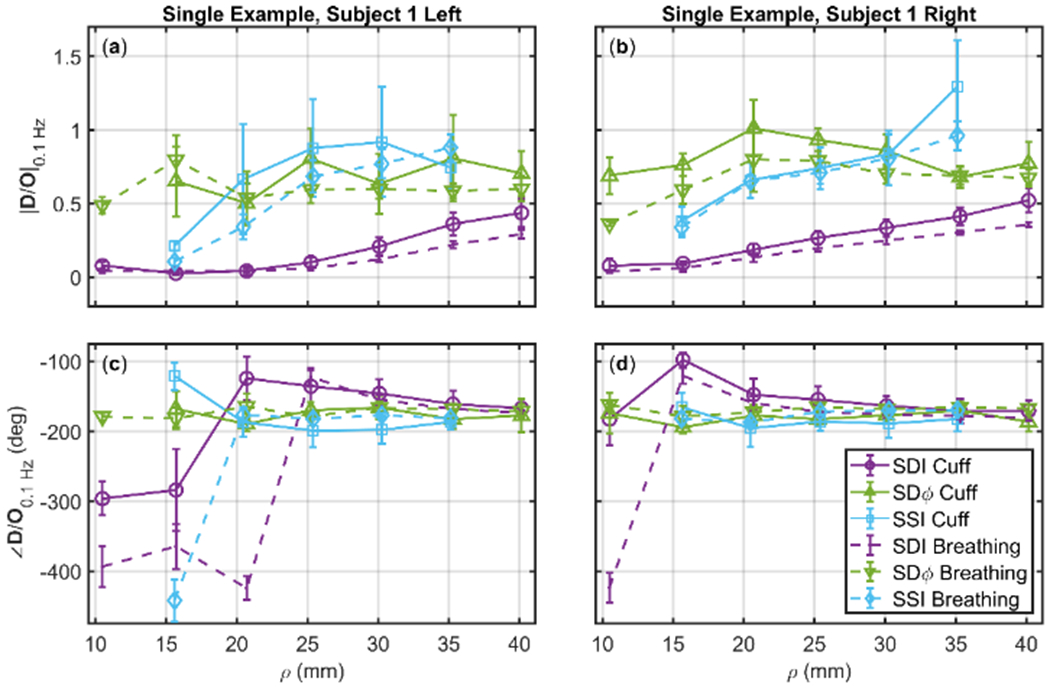
Amplitude ratio ((**a**)(**b**)) and phase difference ((**c**)(**d**)) of Oxyhemoglobin (**O**) and Oeoxyhemoglobin (**D**) phasors at 0.1 Hz as a function of source-detector separation (*ρ*). Data were measured on subject 1 on the left sides ((**a**)(**c**)) and on the right side ((**b**)(**d**)) of the subject’s forehead. Shown for the cyclic thigh cuff inflation (Solid Lines) and paced breathing (Dashed Lines) protocols, and three analysis methods: Single-Distance Intensity (SDI, Purple), Single-Distance phase (SDφ, Green), and Single-Slope Intensity (SSI, Cyan). SSI measurements analyzed with two sources spaced by 10 mm, plotted such that *ρ* is the average of the source-detector distances of the two sources. Note: Error bars represent the standard deviation of all analyzed pixels that show significant coherence in the wavelet transfer function scalogram.

Optical measurements become increasingly sensitive to deeper tissue as the source-detector distance (i.e. the distance between the illumination and collection points) increases. This is true for both Single-Distance (SD) and SS methods. It is also important to consider that intensity and phase data feature different sensitivities to deeper versus superficial tissue. Figure 4 reports the SDI and SDφ measurements at source-detector separations from 11-40 mm, as well as SSI obtained from data at two distances separated by approximately 10 mm (in this case, the data points are assigned to the mean distance in Figure 4) for the amplitude and phase of the phasor ratio **D**/**O**. Data for both protocols, cyclic cuff inflation and paced breathing, are shown in Figure 4 for one representative subject (subject 1) and both sides of the forehead (Figure 4(a)(c): left; Figure 4(b)(d): right). For both protocols, there is a trend of SDI data versus source-detector distance: the amplitude ratio |**D**/**O**| increases from a value <0.1 at short distance, to a value that approaches 0.5 at long distance; the phase difference ∠(**D**/**O**) evolves from an in-phase behavior (−360°) at short distances to an opposition-of-phase behavior (−180°) at long distances.

The results obtained at short distances are consistent with superficial tissue hemodynamics that mostly represent arterial blood volume oscillations (for which **D** and **O** would be in phase, and their amplitude ratio would reflect the oxygen saturation of the oscillating arterial compartment [22]). The results obtained at long distances are consistent with deeper tissue hemodynamics that are characterized mostly by blood flow oscillations (for which **D** and **O** would be in opposition of phase, and would have the same amplitude [22]). In fact, SDφ and SSI data, which are more specifically sensitive to deeper tissue, yield relatively large values (>0.5) for the amplitude ratio |**D**/**O**| and opposition-of-phase (−180°) conditions for the phase difference ∠(**D**/**O**). This point is reinforced by the significantly greater |**D**/**O**| measured with SDφ (35 mm) versus SDI (35 mm) (*p* < 0.0001), and with SSI (30 and 40 mm) versus SDI (35 mm) (*p* < 0.0001). It is worth noting that SDI data (and, to a lesser extent, SDφ data) still show an increasing trend for the amplitude ratio versus distance, whereas the phase difference of ∠(**D**/**O**) is relatively insensitive to the source-detector distance used for SDI and SDφ measurements unless very short distances are considered.

**Figure 5.**
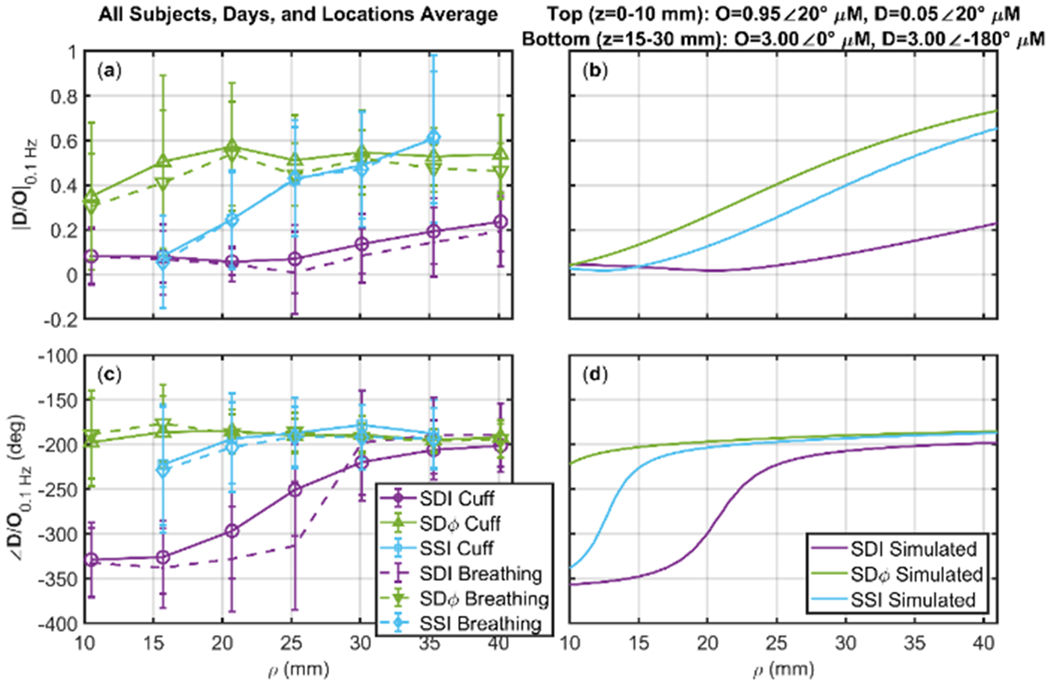
Amplitude ratio ((**a**)(**b**)) and phase difference ((**c**)(**d**)) of Deoxyhemoglobin (**D**) and Oxyhemoglobin (**O**) phasors at 0.1 Hz as a function of source-detector separation (*ρ*). Experimental data in (**a**)(**c**) represent the average over all subjects, days, and locations for cyclic thigh cuff inflation (Solid Lines) and for paced breathing (Dashed Lines). Theoretical calculations in (**b**)(**d**) are for a three-layered medium: top layer from *z*=0-10 mm with **D**/**O**=0.05∠0°; intermediate layer from *z*=10-15 mm with no oscillatory hemodynamics; bottom layer from *z*=15-30 mm with **D**/**O**=1.00∠−180°. Three analysis methods are shown Single-Distance Intensity (SDI, Purple), Single-Distance phase (SDφ, Green), and Single-Slope Intensity (SSI, Cyan). SSI measurements refer to two sources spaced by 10 mm, plotted such that *ρ* is the average of the source-detector distances of the two sources. Note: Error bars show standard deviation of all analyzed pixels that show significant coherence in the wavelet transfer function scalogram across all subjects days and locations.

It is apparent from Figure 4 that SSI and SDφ measurements at source-detector distance of ~20 mm yield values of **D**/**O** that are only obtained at longer distances (>35 mm) with SDI. Particularly for the amplitude of **D**/**O**, it appears that the values obtained with SSI and SDφ at 25 mm represent asymptotic values for SDI (i.e. values that are attained with SDI in the limit of large source-detector separations) that are not reached even at the largest distance considered (40 mm). These results, reported for a single subject in Figure 4, are confirmed by the average over all subjects, shown in Figure 5(a)(c).

To reproduce these results and help interpret them, we have carried out a theoretical calculation, based on diffusion theory, for a three-layered medium structured as follows:

- A top layer (10 mm thick) featuring arterial volume oscillations represented by **D**=0.05∠20° μM and **O**=0.95∠20° μM.
- An intermediate layer (at depths of 10-15 mm) featuring no hemodynamic oscillations.
- A bottom layer (at depths of 15-30 mm) featuring blood flow oscillations represented by **D**=3∠180° μM and **O**=3∠0° μM.

The assumed background absorption coefficient was 0.1 cm^−1^ and the assumed reduced scattering coefficient was 12 cm^−1^. For this three-layered medium, which simulates arterial blood volume oscillations in the scalp, no hemodynamic oscillations in the skull, and flow velocity oscillations in the brain, we computed SDI, SDφ, and SSI data for the same source-detector arrangement of Figure 1(b). The results are shown in Figure 5(b)(d), which largely reproduce our experimental results. From the simulations one may note the significantly greater value of the amplitude of **D**/**O** for SSI and SDφ versus SDI at distances greater than 25 mm, and the shorter distance range (~15-20 mm) at which SSI and SDφ yield the asymptotic value of -180° for the phase of **D**/**O**, which is only approached by SDI at ~35 mm.

### 3.4. Deoxyhemoglobin and Oxyhemoglobin Phasor Ratio (**D**/**O**) Measured at Long Source-Detector Distances with Different Methods

**Figure 6.**
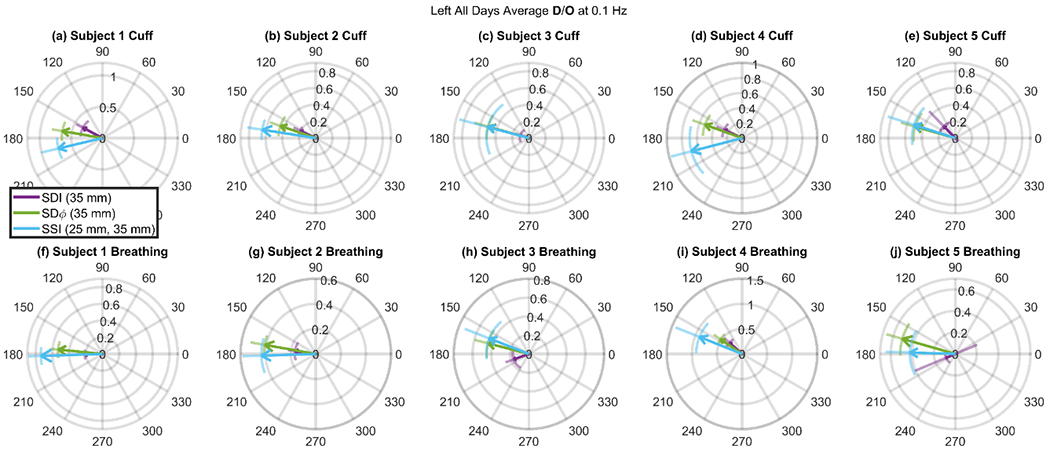
Vectors **D**/**O** whose magnitude and phase represent the amplitude ratio and the phase difference, respectively of the Deoxyhemoglobin (**D**) and Oxyhemoglobin (**O**) phasors at 0.1 Hz. The **D**/**O** vectors are averaged over all days for each subject at one location (left side of the forehead). Error bars are shown as arcs for the phase and line segments extending from the arrow point for the amplitude. Three measurement methods are shown: Single-Distance Intensity (SDI, Purple), Single-Distance Phase (SDφ, Green) and Single-Slope Intensity (SSI, Cyan). (**a**)(**b**)(**c**)(**d**)(**e**) refer to the cyclic cuff inflation protocol, whereas (**f**)(**g**)(**h**)(**i**)(**j**) refer to the paced breathing protocol. Each column (**a**)(**f**), (**b**)(**g**), (**c**)(**h**), (**d**)(**i**), and (**e**)(**j**) refers to one subject, as indicated in the title of each rose. Note: Error bars show standard deviation of all analyzed pixels over all days that show significant coherence in the wavelet transfer function scalogram.

Figure 6 shows **D**/**O** vectors for each individual subject (left side, average over all measurement days) and for both protocols (cuff and paced breathing) measured with SDI (35 mm), SDφ (35 mm), and SSI (25 mm, 35 mm). These long-distance measurements (~25-35 mm) are typically used in cerebral NIRS as it is well established that they achieve a suitable penetration depth to probe the brain. We recall that the amplitude and the phase of **D**/**O** (i.e. the length and direction of the arrows in Figure 6) represent the amplitude ratio and the phase difference, respectively, of the **D** and **O** phasors.

A first result of Figure 6 is that the amplitude of **D**/**O** is consistently and significantly greater when measured with SSI and SDφ (values of ~0.4 or more) than with SDI (values of ~0.2 or less) (*p* < 0.0001). This result suggests that brain tissue (which is most effectively probed with SSI and SDφ versus SDI) features amplitudes of Δ*D* oscillations that approach the amplitude of Δ*O* oscillations.

A second result of Figure 6 is that the phase of **D**/**O** is significantly closer to −180° when measured with SSI or SDφ than with SDI (*p* < 0.0001 for both). By recalling that pure blood flow velocity oscillations (or oscillations in metabolic rate of oxygen) result in Δ*O* and Δ*D* oscillations that are in opposition of phase, this result suggests that brain tissue features blood flow oscillations that dominate over blood volume oscillations.

## 4. Discussion

In this work, we have measured the relative dynamics of the concentrations of *O* and *D* in the tissue probed from the human forehead, in protocols that induce systemic ABP oscillations at a frequency of 0.1 Hz. Our results provide indications on the different depth-sensitivity of three sets of data collected in non-invasive FD NIRS, namely SDI, SDφ, and SSI. It is known that SDI and SDφ have different regions of sensitivity within tissue, with SDφ data probing deeper than SDI, and that SSI affords a reduced sensitivity to the most superficial tissue layer. As expected, SDI data show a trend as the source-detector distance increases, corresponding to the larger contribution from deeper tissue sensed at greater source-detector distances. It is therefore expected that the asymptotic value of SDI data at large distances is more representative of deeper brain tissue, even though it still remains sensitive to extracerebral, superficial tissue. Probably more surprisingly, the SDφ data does not show a similar trend with source-detector distance, or at least SDφ data appear to reach their asymptotic value at a relatively short distance of ~20 mm. We assign this behavior to a significantly greater sensitivity of SDφ data versus SDI data to deeper, cerebral tissue. Similarly, and in this case expectedly, SSI data also shows a greater sensitivity to deeper tissue with respect to SDI data. A major advantage of SDφ data over SSI data is that it only needs one source-detector pair, but it requires the instrumental complexity of FD technology. The increased noise in φ measurements affects even more strongly Single-Slope phase (SSφ) data, not reported here, than SDφ data because of a smaller signal (the phase difference corresponding to Δ*ρ* instead of *ρ*) and a greater noise (because of the need for two φ measurements instead of one). Nevertheless, SSφ holds a significant promise for an even more selective depth sensitivity and should be further explored [24,25].

In terms of the dependence of measured hemodynamics on source-detector distance, the results obtained with the two protocols (cyclic cuff occlusion, paced breathing) are similar (Figure 4, Figure 5). A close inspection of the results obtained at large source-detector separation (Figure 6) shows some differences between the SDI data collected in the two protocols. Typically, ∠(**D**/**O**) is more negative in the cuff protocol than in the paced breathing protocol. If this result is assigned to the brain, a possible explanation is the better cerebral autoregulation during paced breathing, if the subject hyperventilates and therefore creates hypocapnia [32]. However, this difference between protocols is not visible in the SSI data, for which the phase of **D**/**O** vectors are closer and do not show a consistent phase difference between protocols, and even less so in the SDφ data, for which the phase of **D**/**O** vectors is essentially the same for the two protocols. Therefore, we assign the differences between protocols observed in the SDI data to extracerebral tissue contributions, as a result of variable scalp hemodynamic changes in the two protocols. While the relatively small number of subjects (five) in this study does not allow for a statistically significant comparison of the results obtained with the two protocols, we contend that the subjective nature of the paced breathing protocol would introduce a level of variability that is intrinsically linked to the specific subject population. The value of the results reported in this work, which fall short of demonstrating the equivalence or the difference between the two protocols for CHS, lies in confirming the feasibility of both protocols for CHS and in demonstrating a qualitative similarity of the coherent hemodynamics elicited by the two protocols.

We did not observe consistent differences between the right and left sides of the forehead, and across measurement days, at least not within the experimental uncertainties of measured amplitude and phase of **O** and **D** phasors. For this reason, we opted to resort to averages over all subjects, days, and locations in Figure 5(a)(c), and over all days in Figure 6. However, the temporal and spatial variability of oscillatory cerebral hemodynamics in response to controlled systemic perturbations, as studied in CHS, is worth of careful consideration as it may provide valuable functional and physiological information [22]. The spatial dependence of coherent cerebral hemodynamics is an important future direction for CHS imaging.

A key finding of this study is that measurements of the **D**/**O** vector that are more sensitive to brain tissue indicate amplitude of Δ*D* and Δ*O* oscillations that are comparable in amplitude and that approach opposition of phase. This result is consistent with a recent study based on SDI measurements during a cyclic thigh cuff occlusion protocol [33]. A recent hemodynamic model shows that Δ*D* and Δ*O* oscillations are in phase in the case of pure blood volume oscillations (because both *D* and *O* change in the same direction as a result of volume changes), whereas they are in opposition of phase in the case of pure blood flow oscillations (because in the absence of volume changes any increase in *O* must be associated with a decrease in *D* and *vice versa*) [22]. Nevertheless, it is well established that cerebral blood volume changes during brain activation, with increases of the order of 30% as measured with functional magnetic resonance imaging (fMRI) [34,35]. Such blood volume changes appear to be inconsistent with the incompressible nature of brain tissue and its enclosure within the inextensible skull. A possible resolution of this apparent paradox has been proposed by hypothesizing water exchange between capillaries and brain tissue to maintain a constant total tissue volume while expanding the capillary bed [36]. The optical measurements of hemoglobin concentration changes obtained with SDφ and SSI, which are more specific to the brain, may help elucidate the actual cerebral blood volume changes associated with brain activation, hypercapnia, etc.

## 5. Conclusions

In this work, we have compared SDI, SDφ, and SSI data collected with FD NIRS for non-invasive measurements of cerebral hemodynamics. Our results indicate the stronger sensitivity to brain tissue, relative to extracerebral superficial tissue, of SDφ and SSI data with respect to SDI data. A stronger relative sensitivity to deeper versus superficial tissue is of paramount importance in non-invasive brain studies because it suppresses confounding contributions from scalp and skull hemodynamics, thus resulting in more accurate and reliable cerebral measurements. While SSI data improves upon SDI data in terms of specific sensitivity to the brain, SDφ data performs even better, albeit requiring modulated illumination and phase-sensitive detection, and featuring a worse signal-to-noise ratio than SSI data. On the basis of the results presented, we envision an important role for FD φ data (representing the phase of photon-density-waves) in the non-invasive optical study of the brain. When used by itself, i.e. without combining it with intensity measurements, it can be translated into tissue absorption changes similar to the way that the mBLL translates intensity measurements into absorption changes. Such an extension of the mBLL to phase data may enhance the effectiveness of non-invasive optical measurements of the human brain.

## Figures and Tables

**Table 1.** Average baseline absorption coefficients (*μ_a_*) and reduced scattering coefficients ($\mu_{s}^{\prime }$) at 690 nm and 830 nm, Oxy- (*O*), Deoxy- (*D*), and Total (*T*) hemoglobin concentrations, and hemoglobin Saturation (*S*) for the five subjects. Numbers in parenthesis are the standard deviation on the last digit. The hemoglobin parameters are obtained assuming a 70% water volume fraction.

Subject	Gender	Age (years)	*μ_a_*_,690_ *_nm_* (cm^−1^)	*μ_a_*_,830_ *_nm_* (cm^−1^)	$\mu_{s,690nm}^{\prime }$ (cm^−1^)	$\mu_{s,830nm}^{\prime }$ (cm^−1^)	*O* (μM)	*D* (μM)	*T* (μM)	*S* (%)
1	Female	25	0.098(6)	0.106(6)	9.0(5)	7.5(4)	22(3)	18(2)	40(3)	55(4)
2	Female	29	0.10(1)	0.104(5)	10.5(8)	8.9(3)	19(3)	18(2)	37(3)	51(5)
3	Male	29	0.11(1)	0.125(8)	10.0(4)	8.0(1)	28(3)	18(2)	46(4)	60(4)
4	Female	34	0.095(9)	0.092(3)	8.9(5)	6.9(3)	18(2)	17(1)	35(2)	51(3)
5	Male	53	0.12(1)	0.13(1)	10.7(6)	8.6(5)	31(4)	20(3)	51(6)	61(2)

## References

[1] Fantini S; Frederick B; Sassaroli A Perspective: Prospects of non-invasive sensing of the human brain with diffuse optical imaging. APL Photonics 2018, 3, 110901. 31187064 10.1063/1.5038571 PMC6559748

[2] Ferrari M; Quaresima V A brief review on the history of human functional near-infrared spectroscopy (fNIRS) development and fields of application. Neuroimage 2012, 63, 921–935. 22510258 10.1016/j.neuroimage.2012.03.049

[3] Herold F; Wiegel P; Scholkmann F; Müller N Applications of Functional Near-Infrared Spectroscopy (fNIRS) Neuroimaging in Exercise–Cognition Science: A Systematic, Methodology-Focused Review. J. Clin. Med 2018, 7, 466. 10.3390/jcm7120466 PMC6306799 30469482

[4] Nielsen HB Systematic review of near-infrared spectroscopy determined cerebral oxygenation during non-cardiac surgery. Front. Physiol 2014, 5. 24672486 10.3389/fphys.2014.00093 PMC3955969

[5] la Cour A; Greisen G; Hyttel-Sørensen S In vivo validation of cerebral near-infrared spectroscopy: a review. Neurophotonics 2018, 5, 1. 10.1117/1.NPh.5.4.040901 PMC6257082 30525059

[6] Zhang Q; Brown EN; Strangman GE Adaptive filtering for global interference cancellation and real-time recovery of evoked brain activity: a Monte Carlo simulation study. J. Biomed. Opt 2007, 12, 044014. 17867818 10.1117/1.2754714

[7] Saager R; Berger A Measurement of layer-like hemodynamic trends in scalp and cortex: implications for physiological baseline suppression in functional near-infrared spectroscopy. J. Biomed. Opt 2008, 13, 034017. 18601562 10.1117/1.2940587

[8] Gagnon L; Yücel MA; Boas DA; Cooper RJ Further improvement in reducing superficial contamination in NIRS using double short separation measurements. Neuroimage 2014, 85, 127–135. 23403181 10.1016/j.neuroimage.2013.01.073 PMC3665655

[9] Funane T; Atsumori H; Katura T; Obata AN; Sato H; Tanikawa Y; Okada E; Kiguchi M Quantitative evaluation of deep and shallow tissue layers’ contribution to fNIRS signal using multi-distance optodes and independent component analysis. Neuroimage 2014, 85, 150–165. 23439443 10.1016/j.neuroimage.2013.02.026

[10] Franceschini MA; Fantini S; Paunescu LA; Maier JS; Gratton E Influence of a superficial layer in the quantitative spectroscopic study of strongly scattering media. Appl. Opt 1998, 37, 7447. 18301579 10.1364/ao.37.007447

[11] Doulgerakis-Kontoudis M; Eggebrecht AT; Dehghani H Information rich phase content of frequency domain functional Near Infrared Spectroscopy. In Proceedings of the Neural Imaging and Sensing 2019; Luo Q, Ding J, Fu L, Eds.; SPIE, 2019; p. 13.

[12] Lange F; Tachtsidis I Clinical Brain Monitoring with Time Domain NIRS: A Review and Future Perspectives. Appl. Sci 2019, 9, 1612.

[13] Taga G; Konishi Y; Maki A; Tachibana T; Fujiwara M; Koizumi H Spontaneous oscillation of oxy- and deoxy- hemoglobin changes with a phase difference throughout the occipital cortex of newborn infants observed using non-invasive optical topography. Neurosci. Lett 2000, 282, 101–104. 10713406 10.1016/s0304-3940(00)00874-0

[14] Obrig H; Neufang M; Wenzel R; Kohl M; Steinbrink J; Einhäupl K; Villringer A Spontaneous Low Frequency Oscillations of Cerebral Hemodynamics and Metabolism in Human Adults. Neuroimage 2000, 12, 623–639. 11112395 10.1006/nimg.2000.0657

[15] Fenghua Tian; Haijing Niu.; Khan B; Alexandrakis G; Behbehani K; Hanli Liu.; Enhanced Functional Brain Imaging by Using Adaptive Filtering and a Depth Compensation Algorithm in Diffuse Optical Tomography. IEEE Trans. Med. Imaging 2011, 30, 1239–1251. 21296704 10.1109/TMI.2011.2111459

[16] Pierro ML; Sassaroli A; Bergethon PR; Ehrenberg BL; Fantini S Phase-amplitude investigation of spontaneous low-frequency oscillations of cerebral hemodynamics with near-infrared spectroscopy: A sleep study in human subjects. Neuroimage 2012, 63, 1571–1584. 22820416 10.1016/j.neuroimage.2012.07.015 PMC3472105

[17] Sassaroli A; Pierro M; Bergethon PR; Fantini S Low-Frequency Spontaneous Oscillations of Cerebral Hemodynamics Investigated With Near-Infrared Spectroscopy: A Review. IEEE J. Sel. Top. Quantum Electron 2012, 18, 1478–1492.

[18] Watanabe H; Shitara Y; Aoki Y; Inoue T; Tsuchida S; Takahashi N; Taga G Hemoglobin phase of oxygenation and deoxygenation in early brain development measured using fNIRS. Proc. Natl. Acad. Sci 2017, 114, E1737–E1744. 28196885 10.1073/pnas.1616866114 PMC5338505

[19] Reinhard M; Wehrle-Wieland E; Grabiak D; Roth M; Guschlbauer B; Timmer J; Weiller C; Hetzel A Oscillatory cerebral hemodynamics—the macro- vs. microvascular level. J. Neurol. Sci 2006, 250, 103–109. 17011584 10.1016/j.jns.2006.07.011

[20] Wylie GR; Graber HL; Voelbel GT; Kohl AD; DeLuca J; Pei Y; Xu Y; Barbour RL Using co-variations in the Hb signal to detect visual activation: A near infrared spectroscopic imaging study. Neuroimage 2009, 47, 473–481. 19398013 10.1016/j.neuroimage.2009.04.056 PMC7201385

[21] Yoshino K; Kato T Vector-based phase classification of initial dips during word listening using near-infrared spectroscopy. Neuroreport 2012, 23, 947–951. 22989928 10.1097/WNR.0b013e328359833b

[22] Fantini S Dynamic model for the tissue concentration and oxygen saturation of hemoglobin in relation to blood volume, flow velocity, and oxygen consumption: Implications for functional neuroimaging and coherent hemodynamics spectroscopy (CHS). Neuroimage 2014, 85, 202–221. 23583744 10.1016/j.neuroimage.2013.03.065 PMC3760999

[23] Pierro ML; Hallacoglu B; Sassaroli A; Kainerstorfer JM; Fantini S Validation of a novel hemodynamic model for coherent hemodynamics spectroscopy (CHS) and functional brain studies with fNIRS and fMRI. Neuroimage 2014, 85, 222–233. 23562703 10.1016/j.neuroimage.2013.03.037 PMC3740017

[24] Sassaroli A; Blaney G; Fantini S Dual-slope method for enhanced depth sensitivity in diffuse optical spectroscopy. J. Opt. Soc. Am. A 2019, Submitted. 10.1364/JOSAA.36.001743 PMC7160974 31674440

[25] Blaney G; Sassaroli A; Pham T; Fernandez C; Fantini S Enhanced sensitivity to brain tissue with dual-slope cerebral near-infrared spectroscopy. J. Biophotonics 2019, Submitted. 10.1002/jbio.201960018 PMC9219023 31479582

[26] Bigio IJ; Fantini S Quantitative Biomedical Optics; Cambridge University Press: Cambridge, UK, 2016; ISBN 978-0-521-87656-8.

[27] Fantini S; Franceschini MA; Fishkin JB; Barbieri B; Gratton E Quantitative determination of the absorption spectra of chromophores in strongly scattering media: a light-emitting-diode based technique. Appl. Opt 1994, 33, 5204. 20935909 10.1364/AO.33.005204

[28] Fantini S; Hueber D; Franceschini MA; Gratton E; Rosenfeld W; Stubblefield PG; Maulik D; Stankovic MR Non-invasive optical monitoring of the newborn piglet brain using continuous-wave and frequency-domain spectroscopy. Phys. Med. Biol 1999, 44, 1543–1563. 10498522 10.1088/0031-9155/44/6/308

[29] Sassaroli A; Fantini S Comment on the modified Beer–Lambert law for scattering media. Phys. Med. Biol 2004, 49, N255–N257. 15357206 10.1088/0031-9155/49/14/n07

[30] Blaney G; Sassaroli A; Fantini S Algorithm for Determination of Thresholds of Significant Coherence in Time-Frequency Analysis. Biomed. Signal Process. Control 2019, Submitted. 10.1016/j.bspc.2019.101704 PMC9223438 35757281

[31] Hallacoglu B; Sassaroli A; Guerrero-Berroa E; Schnaider Beeri M; Haroutunian V; Shaul M; Rosenberg IH; Toren A; Fantini S Absolute measurement of cerebral optical coefficients, hemoglobin concentration and oxygen saturation in old and young adults with near-infrared spectroscopy. J. Biomed. Opt 2012, 17, 081406. 23224167 10.1117/1.JBO.17.8.081406 PMC3412596

[32] Blaney GP; Krishnamurthy N; Sassaroli A; Pham TT; Fantini S Comparison of spontaneous and induced coherent hemodynamics in the human brain. In Proceedings of the Optical Tomography and Spectroscopy of Tissue XIII; Fantini S, Taroni P, Tromberg BJ, Sevick-Muraca EM, Eds.; SPIE, 2019; p. 54.

[33] Khaksari K; Blaney G; Sassaroli A; Krishnamurthy N; Pham T; Fantini S Depth dependence of coherent hemodynamics in the human head. J. Biomed. Opt 2018, 23, 1. 10.1117/1.JBO.23.12.121615 PMC6318717 30444084

[34] Gu H; Lu H; Ye FQ; Stein EA; Yang Y Noninvasive quantification of cerebral blood volume in humans during functional activation. Neuroimage 2006, 30, 377–387. 16278086 10.1016/j.neuroimage.2005.09.057

[35] Hua J; Liu P; Kim T; Donahue M; Rane S; Chen JJ; Qin Q; Kim S-G MRI techniques to measure arterial and venous cerebral blood volume. Neuroimage 2019, 187, 17–31. 29458187 10.1016/j.neuroimage.2018.02.027 PMC6095829

[36] Krieger SN; Streicher MN; Trampel R; Turner R Cerebral Blood Volume Changes during Brain Activation. J. Cereb. Blood Flow Metab 2012, 32, 1618–1631. 22569192 10.1038/jcbfm.2012.63 PMC3421101

